# Asystole in COVID-19 Infection: A Case Report

**DOI:** 10.7759/cureus.16346

**Published:** 2021-07-12

**Authors:** Umesh Manchandani, Shamsuddin Anwar, Sudeep Acharya, Sakura Thapa, Dany Elsayegh, Mahreen Anwar

**Affiliations:** 1 Internal Medicine, Northwell Health, Staten Island, USA; 2 Pulmonary and Critical Care Medicine, Northwell Health, Staten Island, USA; 3 Biological Sciences, Michigan State University, Lansing, USA

**Keywords:** covid 19, critical care cardiology, internal medicine (general medicine), emerging infections, life threatening arrhythmia

## Abstract

Severe acute respiratory syndrome coronavirus 2 (SARS-CoV-2), the virus that causes coronavirus disease 2019 (COVID-19), has been associated with a broad spectrum of cardiac manifestations ranging from myocardial injury and heart failure to cardiac arrhythmias. In this report, we present a rare case of sinus node dysfunction/asystole in a young patient without any known history of coronary artery disease or cardiac arrhythmias, which necessitated pacemaker placement.

## Introduction

The main systemic illness caused by severe acute respiratory syndrome coronavirus 2 (SARS-CoV-2) infection involves the respiratory tract and sepsis. However, arrhythmias such as myocardial ischemia, QT interval changes, and other EKG changes secondary to electrolyte disturbances can be seen in severe coronavirus disease 2019 (COVID-19), often warranting obtaining a baseline EKG at the time of admission. In this report, we provide an overview of cardiac complications of SARS-CoV-2 infection. We also describe a unique case of prolonged asystole in an otherwise healthy patient, which was caused by SARS-CoV-2 infection, and its possible mechanism.

## Case presentation

A 55-year-old female presented to the hospital after an episode of syncope at home. Her past medical history included Hashimoto’s thyroiditis and hypertension, and both were chronically stable on levothyroxine and an angiotensin receptor blocker. Her current condition had started about seven days prior to the presentation when the patient had developed a dry cough and nasal congestion. She had decided to get tested for SARS-CoV-2 because of her close contact with family members who were positive for SARS-CoV-2 infection. She had turned out to be positive for SARS-CoV-2 infection as well. She had also developed symptoms of progressive episodes of diarrhea. On the day of the presentation, she had been sitting on the toilet seat having a bowel movement when she had suddenly felt her arms and legs becoming weak. She had passed out on the toilet seat and her husband had helped her off the toilet. She denied any head trauma, aura, palpitations, tongue bite, or confusion after the episode of syncope. She had been immediately brought to the hospital for her unwitnessed syncopal episode.

On presentation, her review of systems included subjective fever, shortness of breath on exertion, decreased oral intake, diarrhea, and nausea, but she denied any vomiting, chest pain, and abdominal pain.

**Figure 1:**
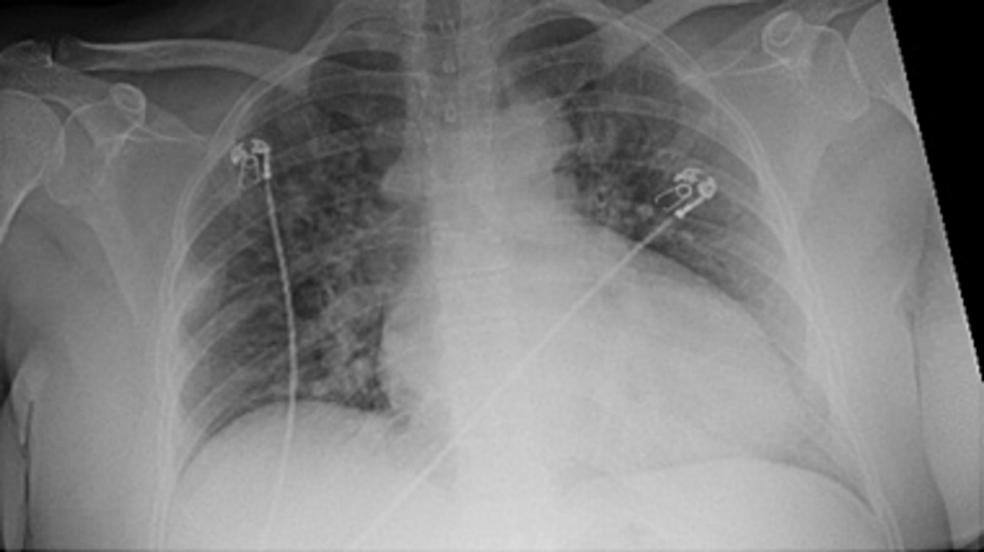
Chest X-ray The imaging shows bilateral small infiltrates consistent with COVID-19. There is no sign of cardiomegaly COVID-19: coronavirus disease 2019

**Figure 2:**
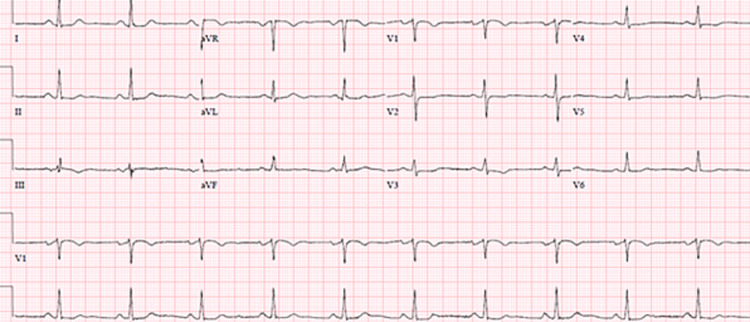
EKG at the time of admission EKG shows normal sinus rhythm EKG: electrocardiogram

The patient tested positive for SARS-CoV-2 on nasal swab again on admission to the hospital. The EKG on admission showed normal sinus rhythm. The vital signs in the emergency department were as follows - temperature: 98.8 °F, heart rate: 60 beats/minute, blood pressure: 106/56 mmHg, respiratory rate: 20 breaths/minute, and pulse oximetry: 93% on room air. She was admitted to the telemetry floor as a part of an evaluation of a suspected syncopal episode. The laboratory results upon admission were as follows - white cell count: 5,860 cell count/uL, hemoglobin: 13.3 g/dL, platelets: 1,36,000 cells/uL, and the complete metabolic panel was unremarkable except for a potassium level of 3.3 mmol/L and blood glucose of 141 mg/dL. The cardiac enzyme (troponin T) was <0.01 ng/ml. Orthostatic vitals on admission were also normal. The inflammatory markers at the time of admission are summarized in Table 1. The patient's chest X-ray is shown in Figure 1, and her baseline EKG is presented in Figure 2.

**Table 1:** Inflammatory markers at the time of admission INR: international normalized ratio

Markers	Results
Ferritin	345 ng/ml
C-reactive protein	16 mg/L
D-dimers	92 ng/mL
Procalcitonin	0.08 ng/mL
Lactate dehydrogenase	299 U/L
Prothrombin time/INR	12.70 seconds/1.10
Activate partial thromboplastin time	36.8 seconds

**Figure 3:**
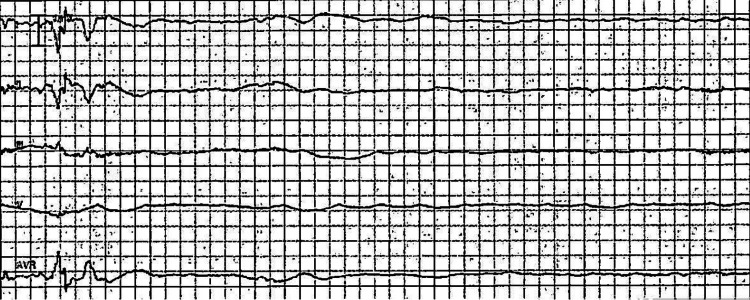
Telemonitor strip Initial rhythm monitor strip during the first episode of asystole

On the third day post-admission, a rapid response was called on her due to acute hypoxia and a brief syncopal episode while she was using the bathroom in the hospital. She was immediately transferred to the bed and evaluated at the bedside. Upon assessment, the patient had regained her consciousness and was oriented to time, place, and person. Her vitals at the time were as follows - heart rate: 62 beats/minute, blood pressure: 153/73 mmHg, but her oxygen saturation had dropped to 88% on the room air, and hence she was placed on a nasal cannula with 4 liters of oxygen per minute. On the telemonitor, she was found to have two consecutive episodes of asystole lasting approximately one minute, 20 seconds, and later one minute, 30 seconds (Figure 3). She was immediately placed on pacer pads and upgraded to the intensive care unit for further evaluation.

The patient was evaluated by the cardio-electrophysiology team for the symptomatic asystole episodes. She was further investigated for thyroid function, cardiac enzymes, and echocardiogram, which were in the normal range. She successfully underwent dual chamber pacemaker placement. For hypoxia, she received remdesivir and steroid treatment as a part of COVID-19 pneumonia treatment. In the later part of the hospital stay, she remained stable and was subsequently discharged home with an outpatient follow-up with the pulmonologist and cardiologist.

## Discussion

COVID-19 infection caused by the SARS-CoV-2 virus is known to present with a wide range of symptoms involving multiple organ systems. While SARS-CoV-2 most commonly affects the respiratory system, cardiovascular events ranging from acute coronary syndrome to cardiac arrhythmias have also been frequently reported [1,2]. The medical literature is continuously evolving with regard to the cardiac complications of SARS-CoV-2 infections in patients with or without prior cardiovascular disease. There is growing evidence from case reports and series about heart failure, acute coronary syndromes, heart blocks, sinus dysfunction, and myocarditis resulting from SARS-CoV-2-related coagulopathies [3]. The exact incidence of cardiac arrhythmias in COVID-19 is not known yet; however, they are estimated to affect 12.9% of the infected population approximately, out of which only 1.2% have developed sinus pauses >3 seconds [4]. We have also taken into account that these reported patients in the medical literature generally had a complicated medical history, as well as complex hospital courses with multiple drugs administered. and hence were already predisposed to develop arrhythmias including sinus pauses.

Cardiac arrhythmias ranging from atrial fibrillation, ventricular fibrillation, and prolonged QTc to bradyarrhythmias including blocks and sinus node dysfunction have been reported in COVID-19 infection [4,5]. These cardiac arrhythmias are usually seen in severe forms of infection and are indicators of poor outcomes [4-6]. The proposed mechanisms of cardiac arrhythmias range from direct myocardial injury, electrolyte imbalances, adverse reactions of medications, hypoxia, and hypercoagulability to systemic effects of pro-inflammatory cytokines [5]. SARS-CoV-2 infection is reported to be associated with hypercoagulability with arterial thromboembolic events [7,8]. Moreover, coronaviruses are also found to spread to the central nervous system, and SARS-CoV-2 has been identified in brain tissue [9].

While most cases of cardiac arrhythmias are noted in patients with a severe form of COVID-19 infection, this article presented a case of a very long episode of asystole in a young patient with a relatively mild form of COVID-19 infection. There are scarce case reports and data in the medical literature about sinus pauses resulting in pacemaker implantation that suggest an association with SARS-CoV-2 infection [10-12]. It is suggested that complications such as cardiac arrests and arrhythmias are likely adverse outcomes of severe systemic illness and not solely the direct effects of SARS-CoV-2 infection [13]. However, our patient had minimal respiratory symptoms on presentation to the hospital, and even during the episode of asystole, she was saturating 88% on room air. EKGs during the hospital course did not show any QTc prolongation. The inflammatory markers were not that significant for severe inflammatory disease. After the workup for other causes of asystole including thyroid function, echocardiogram, and cardiac enzymes, the patient was managed with dual-chamber pacemaker placement and outpatient follow-up with cardiology.

With this unique case report of prolonged asystole in a relatively stable patient, we intend to highlight that the true prevalence of cardiac adverse events in SARS-CoV-2 is not fully appreciated. Further studies will be required to investigate the risk factors and exact mechanisms contributing to the association of SARS-CoV-2 infection with arrhythmias. Furthermore, it is imperative to investigate the effectiveness of vaccination in preventing such serious cardiac complications [14].

## Conclusions

This article reviewed the literature on cardiac arrhythmias in COVID-19 infection and presented a case of asystole in a patient with mild COVID-19 infection. Cardiac arrhythmias are independent markers for the outcome. We recommend that all patients with COVID-19 should be continuously monitored for cardiac events and prompt management of reversible causes to improve overall outcomes.
